# Abiotic Limits for Germination of Sugarcane Seed in Relation to Environmental Spread

**DOI:** 10.1007/s12042-014-9141-9

**Published:** 2014-09-18

**Authors:** J. S. Pierre, A. L. Rae, G. D. Bonnett

**Affiliations:** CSIRO Agriculture Flagship, 306 Carmody Road, St Lucia, Qld 4067 Australia

**Keywords:** Temperature, Water potential, GM crops, Saccharum spp, Biosafety, Caryopsis, Fuzz

## Abstract

Sugarcane is a vegetatively propagated crop and hence the production of seed and its fate in the environment has not been studied. The recent development of genetically modified sugarcane, with the aim of commercial production, requires a research effort to understand sugarcane reproductive biology. This study contributes to this understanding by defining the abiotic limits for sugarcane seed germination. Using seed from multiple genetic crosses, germination was measured under different light regimes (light and dark), temperatures (from 18 °C to 42 °C) and water potentials (from 0 MPa to −1 MPa); cardinal temperatures and base water potential of germination were estimated based on the rates of germination. We found that sugarcane seed could germinate over a broad range of temperatures (from 11 °C to 42 °C) with optima ranging from 27 °C to 36 °C depending on source of seed. Water potentials below −0.5 MPa halved the proportion of seed that germinated. By comparing these limits to the environmental conditions in areas where sugarcane grows and has the potential to produce seed, water, but not temperature, will be the main limiting factor for germination. This new information can be taken into account when evaluating any risk of weediness during the assessment of GM sugarcane.

## Introduction

Sugarcane is a perennial grass cultivated in tropical and sub-tropical regions for the high sucrose content in the culm. The primary use of sucrose is for the food industry but it is also increasingly used for the production of bioethanol (Waclawovsky et al. 2010). In commercial fields, sugarcane plants are obtained by vegetative propagation from pieces of stalk (setts) that are planted and cultivated through multiple ratooning cycles where new shoots grow from their stubbles after harvesting. Contrary to other grass crops, the seed plays no part in the production cycle of sugarcane.

Before the 19th century, the ability of sugarcane to reproduce sexually was unknown and hence for many years, vegetative propagation was the only way to disseminate sugarcane. In 1858, the ability of sugarcane to produce fertile seeds was observed in Java and independently re-observed in Barbados in 1859 (reviewed by Moore et al. 2014). This discovery enabled the start of sugarcane breeding programs in which modern sugarcane varieties (*Saccharum spp.)* were created as hybrids between *Saccharum officinarum* L. (sweet sugarcane) and *Saccharum spontaneum* L. (wild sugarcane) (Buddenhagen 1977). In recent years, sugarcane varietal improvement has reached a new stage with the ability to transform sugarcane to create genetically modified (GM) varieties (Lakshmanan et al. 2005).

In most countries, to ensure protection of environment and human health, the release and cultivation of new GM crops is subject to government regulation (Holst-Jensen 2009). As part of the assessment, the level of change between the parent variety and the GM plants in terms of *spread* and *persistence* is evaluated (Paoletti et al. 2008). Among other things, it is crucial to understand the different means for the plant to spread in its environment (vegetative propagation and/or sexual reproduction) and to assess its weediness potential. It is also important to understand whether the GM trait itself or its pleiotropic effects will modify the extent of spread and persistence.

Since sugarcane is vegetatively propagated, the production of seed and its fate in the environment have not been studied. However, in many sugarcane growing areas sugarcane does flower and is fertile (Berding and Hurney 2005; Bonnett et al. 2010). Consequently, this gap of knowledge needs to be addressed for a complete evaluation of GM sugarcane varieties (Cheavegatti-Gianotto et al*.* 2011; OGTR 2011; OECD 2013). In order to contribute to the environmental risk assessment for GM sugarcane, the effectiveness of different stages of sugarcane sexual reproduction in crop need to be understood including pollen dispersal, ability to cross with other plants, seed physiology, seedling establishment and interaction with the plant community in and around sugarcane fields.

As GM sugarcane varieties are under development, this work has gained importance and in recent years, efforts have been made to study sexual reproduction of sugarcane in commercial fields. It has been demonstrated that in Australian sugarcane growing areas, sugarcane will flower over a wide range of latitudes but will produce viable seed only in an area ranging from approximately latitude 16°S, the northern limit of sugarcane cultivation in Australia, to 20°S (Bonnett et al*.* 2010; Pierre et al., unpublished). In studies reporting the proportion of cross- and self pollination, it was demonstrated that sugarcane is a preferentially out-crossing species (McIntyre and Jackson 2001; Tew and Pan 2010). A theoretical study of the distance to prevent contamination of controlled crosses suggested that the safe distance to prevent sugarcane cross pollination is 100 m in open forest and 300 m in open ground (Skinner 1959). Consequently cross pollination between conventional and GM sugarcane, when grown in the same area, is possible, hence increasing the likelihood to find sugarcane seed with a GM trait outside of the GM fields.

There are no published reports of sugarcane plants establishing from seed in and around sugarcane fields. In North Queensland the weather conditions at the time of seed production, July to August (Olivares-Villegas et al*.* 2010), of low rainfall and temperatures below 25 °C, could be the limiting factors for seed to germinate and seedlings to establish. In Panama (latitude 8°–9°N), *Saccharum spontaneum*, one of the parents of commercial *Saccharum spp.*, is a major weed that establishes from seed (Bonnett et al*.* 2014). There, the consistently higher temperatures and significant rainfall during the seed production period seem to be key factors contributing to this successful invasion.

The fate of fertile sugarcane seeds in major sugarcane production regions in relation to the environmental conditions is largely unknown. Among other things, the cardinal temperatures for seed germination are an important parameter to consider when assessing the likelihood of seed germination in relation to the weather conditions. Previous studies on sugarcane seed germination were aimed at improving the rate of germination for breeding programs, rather than defining the upper and lower limits to germination (Dutt et al*.* 1937; Lee and Loo 1958; Poljakoff-Mayber 1959; Heinz 1974; Itakura et al*.* 1981; Cazalet and Berjak 1983; Singh 1988; Hsu 2001). Where multiple temperatures were tested, the temperatures giving the greatest number of seedlings (not necessarily statistically significant) were 37 °C for *Saccharum spontaneum* L. (Poljakoff-Mayber 1959); 35 °C for one (Itakura et al. 1981) or two sugarcane seed sources (Singh 1988) or 38 °C for 5 seed sources (Heinz 1974).

Whilst these studies have been useful for sugarcane breeding, they do not provide the base line information on the response of germination to a broad range of temperatures that would assist evaluation of GM sugarcane. First, most of the studies used only one or two crosses hence the genetic variation between families was not taken into account. The importance of water availability for seed germination, because it was irrelevant from a breeder perspective, has never been assessed before. Nevertheless for the study of limits to seed germination in relation to environmental conditions, the water availability is as important as the temperature. Furthermore, the term *germination* has been loosely used and does not distinguish between seed germination and seedling elongation, leading to confusion over which phenomenon was observed. Finally observations were made only on the final percentage of germination, and the range of temperatures used was not large enough to define the upper and lower temperature limits of germination.

In this paper we report the first comprehensive study on abiotic limits to germination of sugarcane seed, assessing both temperature and water availability, using seeds from multiple crosses (different parents and year of production) (Table 1). The results define the potential for germination of sugarcane seed and can be compared with the environmental conditions that are likely to be found in sugarcane growing regions at the time of seed production.

**Table 1:** Parents and year of production of the seeds used in the experiment. Q: Queensland (Australia); QC: Queensland Central; QN: Queensland North; H: Hawaii (USA); F: Formosa (Taiwan); CP: Canal Point (USA); VMC: Victoria Milling company (Phillippines). GEMINI was bred in Queensland North

Cross ID	Female parent	Male parent	Year of production
89*758	QC73-214	QN82-115	1989
91*684	Q121	QN84-2850	1991
96*283	H52-663	Q173	1996
98*2	F177	QS80-7059	1998
98*78	QN85-2770	CP67-412	1998
98*148	QN87-2029	QN82-450	1998
98*206	Q174	VMC67-315	1998
98*217	GEMINI	Q162	1998

## Results

### Influence of Light on Germination

When tested for all crosses combined the light conditions did not influence germination (*p-value* = 0.836). However, when the test was performed for seed from each cross individually, six crosses out of eight were not significantly sensitive to light (*p <* 0.05) (Table 2). The germination of seed from two crosses were influenced by the light conditions, either positively (98*78) or negatively (89*758). As overall, light did not appear to influence the germination, subsequent experiments were conducted in the light to help with the frequent observation of germinated seeds.

**Table 2:** Effect of light on seed germination. Sugarcane seeds were incubated at 36 °C in light or dark conditions on wet filter paper for 10 days after which the number of germinated seeds was evaluated. Results are presented as mean ± standard error

	Germinated seed		
Cross	Light	Dark	*p-value*
98*78	37 ± 5	24 ± 1	*0.02*
98*148	112 ± 6	91 ± 10	*0.07*
98*206	60 ± 6	67 ± 5	*0.28*
96*283	47 ± 0	44 ± 5	*0.50*
89*758	28 ± 6	52 ± 8	*0.03*
98*2	44 ± 9	24 ± 6	*0.07*
91*684	56 ± 4	69 ± 6	*0.07*
98*217	44 ± 4	47 ± 2	*0.46*

*P-value* when comparing light versus dark for all crosses combined: 0.836

### Effect of Temperature on Seed Germination

**Fig 1:**
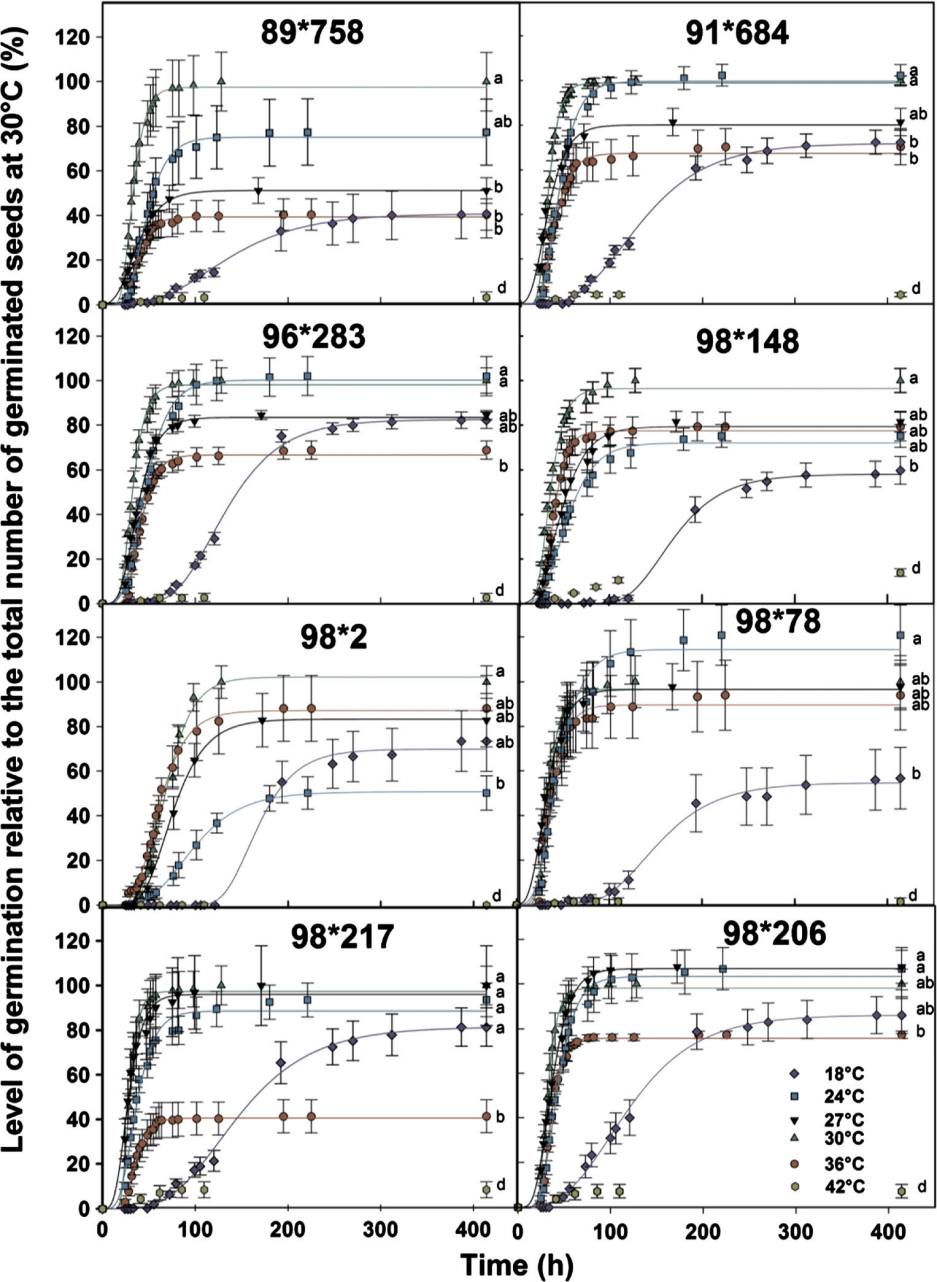
Germination of fuzz of eight sugarcane crosses at different temperature regimes: 18 °C, 24 °C, 27 °C, 30 °C, 36 °C and 42 °C after different periods of time. Symbols represent the experimentally measured cumulative germination percentage relative to the total number of germinated seeds at 30 °C and the lines represent the fitted Gompertz function for each temperature. Error bars denote standard error (±). Letters represent significative differences (*p-value* < 0.05) between final percentages of germination

The effect of temperature on germination was assessed over temperatures ranging from 18 °C to 42 °C. At least some seeds were able to germinate at all temperatures (Fig. 1). At 18 °C the final percentage of germinated seeds ranged from 41 to 82 % while at 42 °C the final percentage of germinated seeds was either null or low (0–13 %) (Fig. 1). At 36 °C, which has been reported to be an optimal temperature for germination, the final number of germinated seeds was significantly lower than at cooler temperatures for five of the eight crosses (Fig. 1).

Seed from all crosses except 98*2 showed a similar trend for their rate of germination. Seeds from cross 98*2 had a slower rate of germination at all temperatures compared to the other crosses (Fig. 1).

**Fig 2:**
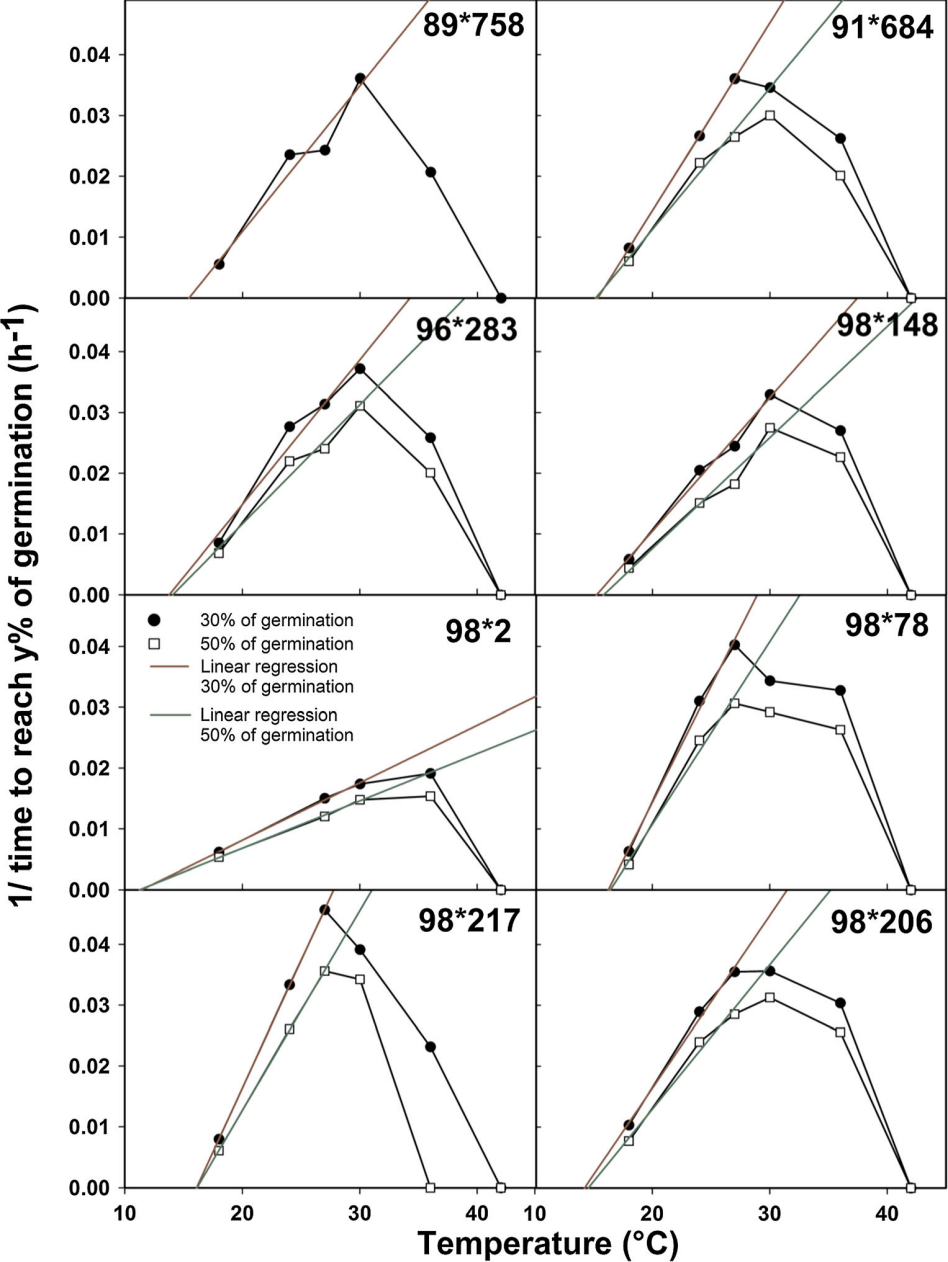
Inverse of the time to reach 30 % (circle) or 50 % (square) of germination as a function of temperature. Straight lines represent the linear relationship between the two factors with the x-intercept being the base temperature of germination

Predicted data obtained from the fitted 3 parameter Gompertz functions were used to estimate the base and optimal temperature of germination as presented in Fig. 2 and Table 3. Because the rate and final percentage of germination was low at 42 °C, this temperature was not included in the analysis. Optimal temperatures of germination ranged from 27 °C to 36 °C (Table 3). Five of the trialled crosses had an optimum temperature for seed germination of 30 °C for the temperatures assessed; 98*2 was the only cross with an optimum of 36 °C, while the remaining two crosses had an optimum temperature of 27 °C.

**Table 3:** Optimum and base temperature of germination of seed from the eight crosses. Estimates of base temperatures of germination were made using the x-intercept resulting from linear regression of the inverse of the time to reach 30 or 50 % of germination as a function of temperature. The r^2^ associated with each linear regression is presented as a superscript

		Base temperature (°C)	
Cross	Optimum (°C)	30 %	50 %
98*2	36	11.4 ^0.998^	11.2^0.998^
98*148	30	15.2 ^0.995^	15.8 ^0.972^
96*283	30	13.7 ^0.995^	14.0 ^0.972^
89*758	30	15.4 ^0.953^	n/a
98*206	30	14.2 ^0.994^	14.6 ^0.985^
91*684	30	15.3 ^1.00^	15.2 ^0.982^
98*78	27	16.2 ^0.996^	16.4 ^0.987^
98*217	27	16.1 ^1.00^	16.1^1.00^

To account for the variability of germination rate between replicates, two estimates of the base temperature of germination were made using the time to reach both 30 and 50 % of germination (Fig. 2). The linear regressions between the inverse of the time to reach 30 or 50 % of germination and temperature were strong with r^2^ values above 0.95. The two estimations of the base temperature of germination were identical or close, varying by less than 0.5 °C, for 6 of the crosses. The difference between the two estimates for 98*148 was 0.6 °C, but according to the r^2^ value the estimate for 30 % of germination of 15.2 °C is more accurate. For 89*758 it was not possible to estimate the base temperature of germination using 50 % of germination since this level of germination was not reached at half of the tested temperatures.

Base temperatures of germination ranged from 11.2 °C to 16.4 °C (Table 3). 98*2 stood out from the other crosses with a relatively lower base temperature of germination of 11.2 °C - 11.4 °C. The other crosses had a base temperature varying between 13.7 °C and 16.4 °C.

At the highest temperature tested, 42 °C, the final number of germinated seed was extremely low and then could not be used to estimate the upper temperature limit for seed germination. For 5 of the crosses (89*758, 91*684, 96*283, 98*2 and 98*78) the final percentage of germination was between 0 and 5 % and for the three other crosses the final percentage of germination ranged between 7 and 14 %. These low numbers of germinated seeds at 42 °C suggest that this temperature is either above or close to the Tmax for germination depending on the crosses.

### Effect of Water Potential on Seed Germination

**Fig 3:**
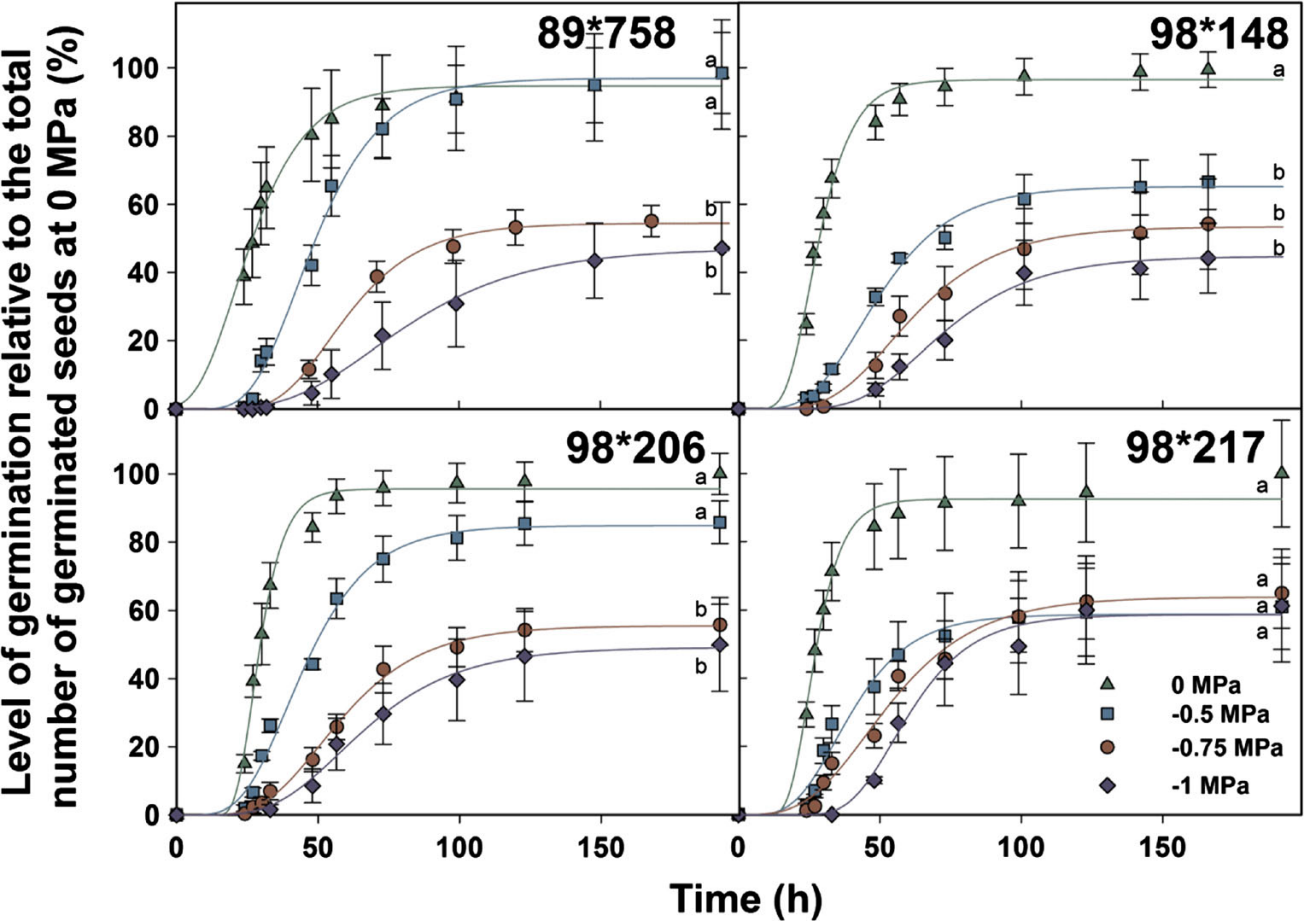
Germination of fuzz of the fourr sugarcane crosses at different water potential regimes: 0 MPa, −0.25 MPa, −0.5 MPa, −1 MPa. Symbols represent the measured cumulative germination percentage relative to the total number of germinated seeds at 0 MPa and the lines represent the fitted Gompertz function for each water potential. Error bars denote standard error (±). Letters represent significative differences (*p-value* < 0.05) between final percentages of germination

The effect of water availability was tested on seed from 4 crosses over a spectrum of water potential ranging from 0 MPa to −1 MPa. The germination rate of all the crosses was affected by the decrease in water potential (Fig. 3). A decrease from 0 to −0.5 MPa doubled the time necessary to reach 30 % germination.

The final percentage of germination of seed from cross 98*148 was the most sensitive to declining water potential (Fig. 3). Seed from crosses 89*758 and 98*206 showed no differences in the final percentage of germination between 0 and −0.5 MPa but then decreased by 50 % at lower water potential. Seed from cross 98*217, showed no statistically significant differences in final percentage of germination between any of the water potential tested (*p-value = 0.06*).

**Fig 4:**
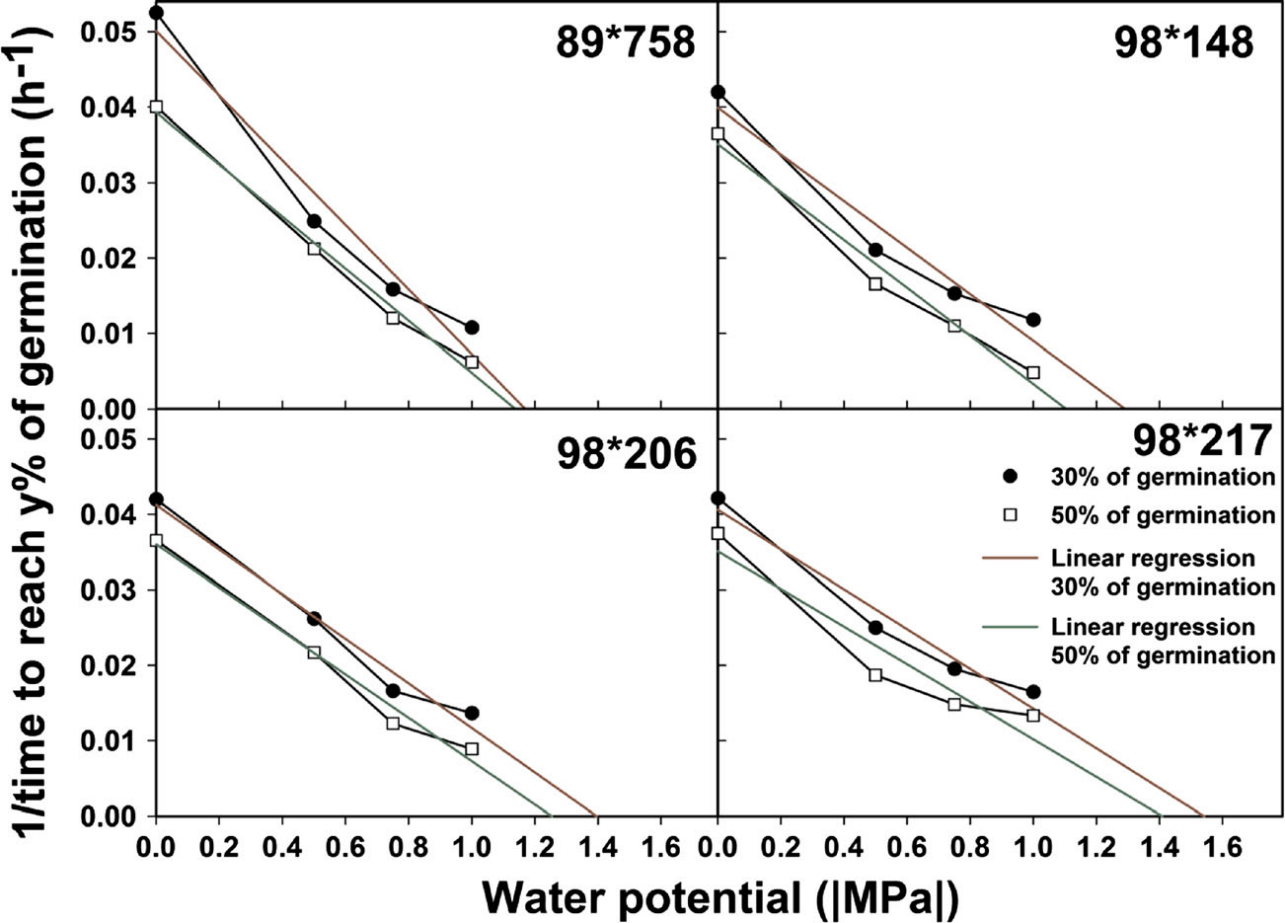
Inverse of the time to reach 30 % (circle) or 50 % (square) of germination as a function of water potential. Straight lines represent the linear relationship between the two factors with the x-intercept being the base water potential of germination

Two estimates of the base water potential of germination were made using the time to reach 30 and 45 % of germination (Fig. 4 and Table 4). The r^2^ values for all relationships were over 0.95 except for 98*217 for time to reach 45 % of germination where r^2^ was 0.91. Overall the differences between the two estimates were between 0.1–0.2 MPa.

**Table 4:** Base water potential for germination of seed from the four selected crosses. Estimates of the base water potential of germination were made using the x-intercept of the linear regression of inverse of the time to reach 30 or 45 % of germination as a function of the temperature. The r^2^ associated with each linear regression is displayed as a superscript

	Base water potential (MPa)	
Cross	30 %	45 %
89*758	−1.2 ^0.964^	−1.1 ^0.992^
98*148	−1.3 ^0.953^	−1.1 ^0.979^
98*206	−1.4 ^0.978^	−1.3 ^0.983^
98*217	−1.5 ^0.962^	*−1.4* ^*0.910*^

Seed from cross 89*758 had the highest sensitivity to decreasing water potential with a base water potential of germination estimated to be between [−1.1, −1.2 MPa] and seed from cross 98*217 has the lowest base water potential of germination [−1.4, −1.5 MPa].

## Discussion

This paper is the first report defining the range of temperatures and water potentials which permit germination of sugarcane true seeds. By comparison with long-term meteorological records, this information can help to identify geographical regions and climatic zones in which seeds can germinate in the environment. This characterization of the biological limits for sugarcane seed germination will serve as a reference against which potential changes in a GM variety could be compared if necessary.

In this study, the materials and the treatment conditions have been chosen to be relevant to the experienced environment in places where sugarcane grows. Temperatures and water potentials were selected within the range of conditions that may occur in sugarcane growing regions. The selected sources of seed came from eight different bi-parental crosses with 16 different parents. The results therefore encompass part of the genetic diversity that exists in sugarcane breeding germplasm. The previous studies about sugarcane seed germination had always used seeds coming from small number of varieties therefore reducing the possible impact of genetic variation on seed germination (e.g. Singh et al*.* 1988).

Sugarcane varieties are highly heterozygous polyploids and hence within an inflorescence, the seeds are not genetically identical. In addition the highly complex nature of the sugarcane genome leads to chromosome loss during meiosis (Cuadrado 2004) which increases the genetic variability between individual seeds. Therefore some of the variability observed between replicates in our experiments is the result of genetic diversity between seeds from the same cross.

To mimic natural conditions, fuzz instead of bare caryopsis, was used for our experiments as the caryopses normally remain tightly attached to the floret structures when florets drop from the inflorescence at maturity. The floret structures, lemma, palea and glumes, are known to restrict oxygen diffusion and to limit water movement (Simpson 1990) and increases in seed germination in dormant and non-dormant seed have been demonstrated when the caryopsis was separated from these structures (Simpson 1990). Therefore removing them may lead to different conclusions regarding the water requirement for seed germination.

Significant numbers of seeds germinated at the lowest temperature tested (18 °C) and the estimated base temperatures of germination varied between 11.2 °C and 16.4 °C. These results set a lower limit to germination temperature that is 10 °C to 15 °C below the temperature that was reported to strongly inhibit germination (Heinz 1974; Itakura et al*.* 1981; Singh 1988). In regions of Australia where fertile seed is produced in commercial fields, (latitudes S16° to S20°, Bonnett et al. 2010; Pierre et al., unpublished) the mean minimal temperature during winter does not fall below 18 °C (source: Bureau of Meteorology 2014). With on average 69 % of seeds still able to germinate at 18 °C, temperature is not a limitation for sugarcane seed germination at those latitudes. Similar analyses are presented for the countries that are the largest producers of sugarcane. In Brazil the main sugarcane producing area is around São Paulo where the temperatures during the coldest month of the year do not fall below 15 °C (source: World Bank 2014). In the northern part of the country, close to Maceió (Alagoas state) where sugarcane breeding also takes place and where the production of fertile seeds is more likely, temperatures don’t fall below 24 °C. In the southern state of Maharashtra, one of the major areas of production for sugarcane in India, the mean minimal temperatures between Solapur (N17°) to Aurangabad (N19°) are always above 20 °C (source: World Bank 2014) and in the area of Coimbatore (N11°) where sugarcane is bred, the minimal temperatures are around 24 °C. Finally in China, in the areas of Kaiyuan (N23°) and Guangzhou (N23°), where two of the Chinese sugarcane breeding institutes are located, the mean minimal temperatures are 10 °C and 13 °C respectively, reducing greatly the possibility of sugarcane seed to germinate. Except for China, if water is not a limiting factor, it is likely that a proportion of seed from some genotypes would be able to germinate under these conditions and this proportion is likely to increase with decreasing latitude. The newly defined base temperature of germination will be important data for assessing the regulation of GM sugarcane. In fact, with only the previous reports on sugarcane seed germination showing a relatively high temperature requirement for optimal germination (~36 °C) (Heinz 1974; Itakura et al*.* 1981; Poljakoff-Mayber 1959; Singh 1988) there was an implied impediment for their germination at the time of their production in the field.

The optimal temperature of germination determined by this study is lower than previously published work in sugarcane. The literature on sugarcane seed germination reports an optimal temperature of germination around 37 °C for *Saccharum spontaneum* (Poljakoff-Mayber 1959); and 35 °C to 38 °C for *Saccharum spp.* (Heinz 1974; Itakura et al*.* 1981; Singh 1988). For seed from 7 of the 8 crosses, the optimal temperature of germination was significantly lower, ranging from 27 °C to 30 °C. We demonstrated that 36 °C is a supra optimal temperature for germination that leads to a slower rate of germination and a decreased number of germinated seeds for a majority of the samples tested. These results are of importance to sugarcane breeding programs, where seedlings are frequently germinated at 35 °C (Breaux and Miller 1987). Use of lower temperatures should be investigated to test whether it improves both the rate and number of seedlings raised.

In tropical cereals where the seed is the basis of propagation and production, the conditions that allow seed germination are well defined. The cardinal and optimal temperatures for seed germination of the main tropical cereal crops are 6 °C/43 °C/34 °C for maize (Itabari et al*.* 1993), 8.5 °C/49 °C/35 °C for sorghum (Harris et al*.* 1987); 10 °C/45 °C/30 °C for rice (Yoshida 1981) and 12 °C/48 °C/34 °C for pearl millet (Garcia-Huidobro et al*.* 1982). Sugarcane has a base temperature of germination which is higher than these tropical grass crops and a maximal temperature of germination which we assume to be close to the one for maize (>42 °C). It is likely that selection for efficient seed germination in the tropical cereals has resulted in a broader adaptation to climatic conditions than has occurred in sugarcane, which has not undergone any selection for seed traits. Base temperatures for other developmental stages of sugarcane have been defined and it appears that vegetative sett “germination” is less sensitive to cold than seed germination with a base temperature around 10.8 °C-12.7 °C (Humbert 1968; Yang and Chen 1980; Donaldson 2009).

The availability of water is a key factor in seed germination. Low water potential will slow down the germination process due to an increased difficulty for the seed to extract water from its surrounding media. Depending on the species and the intensity of the water deficit, it will inhibit or greatly compromise germination. In our experiments we have shown diverse response of sugarcane seed germination to decreasing water potential. For three of the tested crosses the final percentage of germination was not significantly affected by mild water deficit (−0.5 MPa). Except for 98*217, subsequent decrease in water potential impacted significantly the final number of germinated seeds reducing it by nearly half. The heterogeneous response to decreasing water potential between the crosses results in a wide range of base water potential of germination estimated between −1.1 and −1.5 MPa. As a comparison, bud outgrowth from setts and subsequent root elongation is significantly reduced when soil water potential equals −0.5 MPa and bud outgrowth is minimal between −2.0 to −3.0 MPa. (Singh and Srivastava 1974). In regards to other cereal crops, sugarcane germination response to water deficit is close to that observed for sorghum (Stoutt et al*.* 1980) and barley (Zhang et al*.* 2010). For sorghum, the ability to germinate under water deficit is highly dependent on the variety. Some varieties will achieve 100 % of germination at −0.6 MPa while at this same water potential other varieties will barely reach 20 %. For barley, the final percentage of germination at −0.9 MPa was 70–73 % and then dropped dramatically at lower water potential. By contrast wheat seeds will achieve a high level of germination for soil water potential values that are below sugarcane permanent wilting point of −1.5 MPa (Inman-Bamber et al*.* 1998) with more than 75 % of germination at −1.6 MPa (Wuest and Lutcher 2013; Hampson and Simpson 1990).

Where fertile sugarcane seeds are produced in Australia, it happens during the driest time of the year with monthly rainfall between 40–70 mm and mean maximal temperature around 26 °C (source: Bureau of Meteorology 2014). This combination of low rainfall and high temperature favours drying of the soil surface. In such environments seed germination and subsequent root elongation needs to happen quickly for the root to reach soil layers less depleted in water to supply water demand for seedling establishment. Our results show that decreasing water potential considerably slows down the germination process and for most crosses water potentials below −0.5 MPa greatly affect the final percentage of germinated seeds. Therefore water availability at the time of seed ripening and release seems to be the main limiting factor for seed germination in sugarcane growing areas where viable seed are produced. Nonetheless, in other parts of the world water availability at the time of seed setting is not a limiting factor. For example, in Panama (latitude 6°N-7°N) *Saccharum spontaneum*, one of the progenitor species of modern sugarcane, is a weed of major importance (Saltonstall and Bonnett 2012). Its flowering and seed setting occur during a time of the year where monthly average rainfall is between 300 mm to 500 mm (source: World Bank 2014) resulting in massive populations that the genetic evidence suggests are growing from seeds (Bonnett et al*.* 2014).

In conclusion, our data provide important information about sugarcane reproductive biology which will assist the scientific evidence-based regulation of genetically modified sugarcane. Whilst temperature does not appear to be a limiting factor in most areas where sugarcane is grown, the water availability at the time of seed production seems to be a major limiting factor for germination. Therefore, water availability at the time of seed production needs to be taken into account to evaluate the likelihood of GM cane establishment from seed. A further key question will be whether seed can persist in the environment until periods of increased rainfall occur.

## Material and Methods

### Seed Material

Seed (caryopsis) was sourced from eight bi-parental sugarcane crosses made at Meringa (Sugar Research Australia (SRA), formerly BSES Ltd.) (17°3′52.63″S, 145°46′27.72″E) in northern Queensland, between 1989 and 1998 (Table 1), with parents originating from a range of countries (Table 1). Breeder cross material was used because it provides large quantity of highly fertile material which is not possible to obtain from field samples.

After harvesting the inflorescence, the fuzz (a mixture of sterile and fertile florets) was dried for 5 days at 12 °C and 10 % humidity then packed in sealed polypropylene bags and stored at −20 °C. In December 2012, bags were transferred to a cold room at 3 °C at 31 % relative humidity.

Sugarcane seed is very small (approximately 1.8 mm × 0.8 mm) and tightly enclosed in bracts (Cheavegatti-Gianotto et al*.* 2011, De Siqueira et al.*,* submitted). In our experiments, to mimic natural conditions, seeds were not separated from the fuzz and fuzz was not sterilized for the germination experiments. The amount of fuzz put into each box was determined through a germination test conducted at 36 °C with and without light (*n* = 5) and corresponded to an average number of 30 to 60 germinated seeds per replicate (Table 2).

### Influence of Light on Seed Germination and Early Growth

Light can influence germination of seeds (Frankland and Taylorson 1983). For sugarcane hybrids there are no statistically analysed comparisons of germination in the light and dark, though Itakura et al. (1981) suggested that light improved germination. Consequently we tested the effect of light on the germination of the eight seed sources. Fuzz (0.3 g) was evenly spread onto filter paper (Whatman, filter paper 3, 125 mm) within large Petri dishes (Corning, 150 mm × 25 mm). The dark treatment was watered, with 20 ml of distilled water, and wrapped in aluminium foil in a dark room under red light of low intensity. The light treatment was also watered with 20 ml of distilled water. The edge of the dishes was sealed with a strip of aluminium foil to ensure that the humidity was the same for both treatments. Petri dishes were then placed in a random order in an incubator (Sanyo, MLR 350HT) at a constant temperature of 36 °C with light (198 ± 34 μmol photons m^−2^ s^−1^). After 10 days the number of germinated seed was assessed.

### Influence of Temperature and Water Potential on Seed Germination

In each experiment, 0.3 g of fuzz was used with the exception of cross 98*148 where 0.15 g of fuzz was used. Fuzz was well spread onto filter paper laid down into large Petri dishes (as described above). Dishes were watered with 20 ml of distilled water, placed in zip-lock bags containing a wet paper towel to maintain a water saturated atmosphere and then placed in an incubator at the required temperature in constant light (198 ± 34 μmol photons m^−2^ s^−1^).

The influence of temperature on sugarcane seed germination at 18 °C, 24 °C, 27 °C, 30 °C, 36 °C and 42 °C was tested. Observation on the number of germinated seeds (visible protrusion of the radicle) were carried out commencing 24 h after incubation, initially every 3 h, then less frequently, until no further germination was observed. The relative percentage of germination of seed from each cross was calculated at each temperature using the mean final number of germinated seed at 30 °C as 100 % which corresponded to actual numbers of germinated seeds for each cross of: 59 ± 8 (89*758), 78 ± 2 (91*684), 79 ± 5 (96*283), 81 ± 4 (98*148), 49 ± 4 (98*2), 70 ± 4 (98*206), 59 ± 5 (98*217), 27 ± 3 (98*78).

The influence of water potential on seed germination at 0, −0.5, −0.75 and −1 MPa was tested at 30 °C under constant light (198 ± 34 μmol photons m^−2^ s^−1^) for four crosses (89*758, 98*148, 98*206, 98*217). Water potentials were generated with PEG 8000 (Sigma-Aldrich) according to Michel (1983) and verified using an osmometer (Vapro osmometer 5520, Wescor).

For each cross the relative percentage of germination was calculated compared to the mean final number of germinated seeds at 0 MPa.

### Determination of the Base and Optimal Temperature and Water Potential for Seed Germination

The germination rates (mean of 5 replicates) at each time point for each of the different temperatures or water potentials were fitted to a 3 parameter Gompertz function:

$$ f(t)=a{e}^{-{e}^{\left(x-c\right)/b}} $$

The resulting a, b and c parameters were used to calculate the time to reach different cumulative germination values as described in Brunel et al. (2009). For each treatment, we selected two cumulative germination values (y%) within the exponential phase of germination: 30 and 50 % for the temperature treatment and 30 and 45 % for the water deficit treatment. For each treatment level, the time to reach these cumulative germination values was calculated and used to generate the graph of 1/time to y% of germination as a function of treatment levels. The base temperature and base water potential of germination were obtained from the x-intercept of the linear regression between these two factors when the linear relationship was strong (r^2^ > 0.95) (Dahal and Bradford 1994).

For the temperatures tested, the optimal temperature of germination was defined as the temperature at which the time to reach 50 % of germination was the smallest.

### Statistical Analyses

Two sided t-tests were performed to test for significant effect (*p <* 0.05) of light on seed germination. Differences in the final number of germinated seeds for the temperature and water potential experiments were assessed by one way analysis of variance and pair wise multiple comparison with Holm-Sidak post hoc analysis used to compare the final number of germinated seeds between temperatures or water potentials.

## Abbreviations

GM: Genetically modified
